# Dietary Compound Chrysin Inhibits Retinal Neovascularization with Abnormal Capillaries in db/db Mice

**DOI:** 10.3390/nu8120782

**Published:** 2016-12-03

**Authors:** Min-Kyung Kang, Sin-Hye Park, Yun-Ho Kim, Eun-Jung Lee, Lucia Dwi Antika, Dong Yeon Kim, Yean-Jung Choi, Young-Hee Kang

**Affiliations:** Department of Food Science and Nutrition, Hallym University, Chuncheon 24252, Korea; mitholy@hallym.ac.kr (M.-K.K.); sinhyepark@hallym.ac.kr (S.-H.P.); royalskim@hallym.ac.kr (Y.-H.K.); reydmswjd@naver.com (E.-J.L.); lucia.dwiantika@gmail.com (L.D.A.); ehddus3290@naver.com (D.Y.K.); yjchoi79@hallym.ac.kr (Y.-J.C.)

**Keywords:** angiogenesis, chrysin, diabetic retinopathy, retinal neovascularization, blood retinal barrier

## Abstract

Diabetic retinopathy (DR) develops in a significant proportion of patients with chronic diabetes, characterized by retinal macular edema and abnormal retinal vessel outgrowth leading to vision loss. Chrysin, a naturally-occurring flavonoid found in herb and honeycomb, has anti-inflammatory, antioxidant, and anti-cancer properties. This study sought to determine the protective effects of chrysin on retinal neovascularization with abnormal vessels and blood-retinal barrier (BRB) breakdown in 33 mM glucose-exposed human retinal endothelial cells and in db/db mouse eyes. High glucose caused retinal endothelial apoptotic injury, which was inhibited by submicromolar chrysin. This compound diminished the enhanced induction of HIF-1α, vascular endothelial growth factor (VEGF), and VEGF receptor-2 (VEGFR2) in high glucose-exposed retinal endothelial cells. Consistently, oral administration of 10 mg/kg chrysin reduced the induction of these proteins in db/db mouse eye tissues. In addition, chrysin restored the decrement of VE-cadherin and ZO-1 junction proteins and PECAM-1 in hyperglycemia-stimulated retinal endothelial cells and diabetic mouse retina, possibly maintaining tight cell-cell interactions of endothelial cells and pericytes. Anti-apoptotic chrysin reduced the up-regulation of Ang-1, Ang-2, and Tie-2 crucial to retinal capillary occlusion and BRB permeability. Furthermore, orally treating chrysin inhibited acellular capillary formation, neovascularization, and vascular leakage observed in diabetic retinas. These observations demonstrate, for the first time, that chrysin had a capability to encumber diabetes-associated retinal neovascularization with microvascular abnormalities and BRB breakdown.

## 1. Introduction

Diabetic retinopathy (DR), known as diabetic eye disease, results in microvascular retinal changes eventually leading to visual impairment or blindness [1,2]. Hyperglycemia evokes intramural pericyte apoptosis and basement membrane thickening, ultimately causing the dysfunction of blood-retinal barrier (BRB) and makes retinal vessels leaky [3,4,5]. Diabetic macular edema and blurry vision occur when the damaged vessels leak blood, fluid exudates, and lipids into the macula and retina [6,7]. Hyperglycemia-associated retinal neovascularization forming abnormal new blood vessels can accompany retinal and vitreous morphological changes [6,8]. Aberrant angiogenesis of retinal vessels that are often immature results in vitreous hemorrhage, increased vascular permeability, fibrovascular tissue formation, and traction retinal detachments leading to vision loss [9]. Retinal ischemia, an ocular manifestation of diabetes, occurs owing to narrowing or blocked retinal blood vessels [10,11]. Retinal neovascularization occurs in ischemic retinopathies in which damage to retinal vessels results in retinal ischemia [10,12]. In addition, hypoxia has been implicated as a causative factor in the retinal degeneration induced by hyperglycemia, possibly contributing to pathological neovascularization and vascular dystrophies with vitreous hemorrhage induction [11,13,14]. 

Pathophysiological mechanisms triggered by hyperglycemia in the development of DR include genetic and epigenetic factors, oxidative stress, advanced glycosylation end products, and inflammatory mediators [6]. Inflammation has been reported to mediate structural and molecular alterations associated with DR [15]. However, the molecular mechanisms underlying the inflammatory pathways associated with DR are still not defined. There is convincing evidence that reactive oxygen species (ROS) promote apoptosis of human lens epithelial cells, vascular cells, and neuronal cells and stimulate inflammation and abnormal angiogenesis in the DR process [11,16]. Hyperglycemia induces several alterations including leukostasis, vasoconstriction and a pro-inflammatory state that causes hypoxia in the retina, but not necessarily via ROS [14]. In addition, chronic and sustained hyperglycemia that culminates in vascular dysfunction results in the accumulation of advanced glycation end products, the increment of growth factors such as vascular endothelial growth factor (VEGF) and consequent leakage of capillary endothelial cells [13,14]. During DR progression, inflammation, and angiogenesis mutually contribute to vasopermeability and/or pathological angiogenesis through enhancing the production of pro-inflammatory mediators and VEGF due to tissue hypoxia [13]. 

Various pro-angiogenic factors have been shown to play major roles in the development of retinal neovascular vessels [9]. Stabilization of hypoxia inducible factor-1 (HIF-1) leads to up-regulation of several hypoxia-regulated gene products including VEGF, angiopoietin (Ang)-2, and vascular endothelial-protein tyrosine phosphatase, causing further stimulation of the neovascularization [12,17]. Stimulation of VEGF signaling and suppression of Tie-2 by Ang-2 are critical for developing retinal neovascularization and vascular leakage [18]. However, there is no clear-cut evidence for the crosstalk between VEGF and Ang-2 to promote vascular permeability. Chronic retinal microvascular damage results in the elevation of intraocular levels of VEGF that mediates diverse physiologic processes, as well as the development and permeability of the vasculature [19]. Thus, anti-angiogenic neutralization of VEGF by anti-VEGF agents is currently the first line therapy and may be beneficial in the treatment of vascular leakage, macular edema, and neovascular glaucoma [17,19,20]. In addition, new targets such as Tie-2 stabilization are possible for the additional treatments of retinal vascular complications [21]. There is a significant body of emerging the evidence that the interacting actions of Ang-1 with Tie-2 are of potential benefit in pathologies of vascular activation of DR [21,22]. Potentially deleterious consequences of administering Ang-1 for prolonged periods include unwanted angiogenesis and potential promotion of tumors [21]. Several novel intravitreal medications and dexamethasone implants have provided promising therapeutic options [23,24].

**Figure 1:**
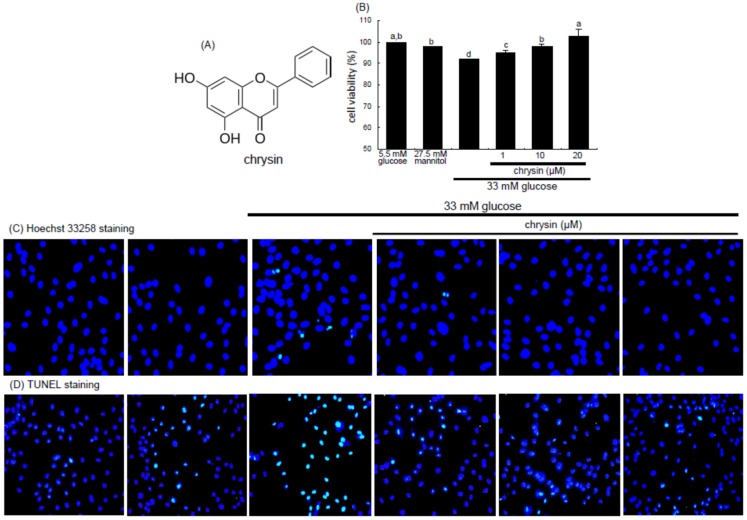
Chemical structure of chrysin (**A**); inhibitory effects of chrysin on viability (**B**); nuclear condensation (**C**); and DNA fragmentation (**D**) in and glucose toxicity of retinal endothelial cells. human retinal microvascular endothelial cells (HRMVEC) were incubated with 33 mM glucose in the absence and presence of 1–20 μM chrysin up to five days. Cells were also incubated with 5.5 mM glucose and 27.5 mM mannitol as osmotic controls. After HRMVEC were cultured in media for two days, cell viability was measured by using MTT assay (*n* = 3, 100% viability with 5.5 mM glucose). Means not sharing a common letter differ, *p* < 0.05. Nuclear condensation was examined with Hoechst 33258 in HRMVEC (**C**) and the DNA fragmentation measured with TUNEL assay and nuclear staining was done with 4′,6-diamidino-2-phenylindole (DAPI) (**D**). Representative microphotographs were obtained by fluorescent microscopy with fluorescein green filter. Magnification: 200×. Fluorescence intensity was quantified by using an Axiomager (Zeiss, Oberkochen, Germany). microscope system. Respective values not sharing a letter are different at *p* < 0.05.

Numerous anti-angiogenic compounds have been suggested as potential therapeutic agents in the milieu of ocular neovascular diseases [25]. However, anti-VEGF agents may cause ocular or systemic side effects after intraocular administration. Natural products have been developed as anti-angiogenic agents with minimal side effects, with a specific focus on retinal and choroidal neovascularization [25]. However, they have not been proven effective in randomized, controlled trials. Natural products may serve as specific, efficacious, and affordable agents for a potential DR therapy [26,27,28]. Epigallocatechin-3-gallate (EGCG) inhibits ocular neovascularization and vascular permeability via suppression of MMP-9 and VEGF activation [26]. The flavone scutellarin shows potential in inhibiting retinal angiogenesis through ROS/HIF-1α/VEGF pathway [27]. Chrysin (Figure 1A) is a flavone-type flavonoid found in honey and herbs and possesses diverse pharmacological activities and multiple biological effects in the central nervous and immune systems [29,30]. Our previous study showed that the multifunctional chrysin exhibited the renoprotection by inhibiting diabetic renal tubulointerstitial fibrosis through blocking epithelial to mesenchymal transition [31]. However, whether chrysin maintains eye health in diabetes remains elusive. The current study attempted to determine whether chrysin antagonized high glucose-induced angiogenic effects in retinal microvascular endothelial cells and inhibited retinal neovascularization in mouse diabetic models.

## 2. Materials and Methods

### 2.1. Materials

Fetal bovine serum (FBS), trypsin-EDTA, and penicillin–streptomycin were purchased from Lonza (Walkersvillle, MD, USA). M199 media, mannitol, and d-glucose were obtained from Sigma-Aldrich Chemical (St. Louis, MO, USA), as were all other reagents, unless specifically stated elsewhere. Antibodies of mouse monoclonal HIF-1α, rabbit polyclonal vascular endothelial cadherin (VE-cadherin), and rabbit polyclonal neural cadherin (*N*-cadherin) were supplied by Abcam Biochemicals (Cambridge, UK). Goat polyclonal antibodies of zona occluden (ZO)-1, platelet endothelial adhesion molecule (PECAM)-1, Ang-1, Ang-2, and Tie-2 were obtained from Santa Cruz Biotechnology (Santa Cruz, CA, USA). Goat polyclonal human/mouse VEGF antibody was provided by R and D systems (Minneapolis, MN, USA), and rabbit monoclonal VEGF receptor-2 (VEGFR2) antibody purchased from Cell Signaling Technology (Beverly, CA, USA). Rabbit polyclonal phospho-Tie-2 antibody was provided by Millipore Corporation (Temecula, CA, USA). Horseradish peroxidase (HRP)-conjugated goat anti-rabbit immunoglobulin (Ig)G, goat anti-mouse and donkey anti-goat IgG were purchased from Jackson ImmumnoReserch Laboratories (West Grove, PA, USA). Essentially fatty acid free bovine serum albumin (BSA) and skim milk were supplied by Becton Dickinson Company (Sparks, MD, USA). Chrysin was dissolved in dimethyl sulfoxide (DMSO) for live culture with cells; a final culture concentration of DMSO was <0.5%.

### 2.2. Retinal Endothelial Cell Culture

Primary human retinal microvascular endothelial cells (HRMVEC) were obtained from Cell System Corporation (Kirkland, WA, USA). Cells were grown in M199 medium containing 10% FBS, 100 U/mL penicillin, 100 μg/mL streptomycin, 2 mM glutamine, 0.75 μg/mL human epidermal growth factor, and 75 μg/mL hydrocortisone at 37 °C humidified atmosphere of 5% CO_2_ in air. HRMVEC were sub-cultured at 90% confluence and used for further experiments within 10 passages. To induce angiogenesis, HRMVEC were incubated in media containing with normal glucose (5.5 mM d-glucose), 27.5 mM mannitol as an osmotic control or high glucose (33 mM d-glucose) in the absence and presence of 1–20 μM chrysin for 48–72 h.

The HRMVEC viability was determined by assaying with MTT (3-(4,5-dimethylthiazol-2-yl)-2,5-diphenyltertrazolium bromide). HRMVEC seeded at a density of 1 × 10^4^ cells/mL on a 24-well plate were treated with 1–20 μM chrysin in different glucose media. Cells were incubated with 1 mg/mL MTT solution at 37 °C for 3 h, forming insoluble purple formazan product that was dissolved in 250 μL isopropanol. Optical density was measured using a microplate reader at the wavelength of 570 nm. Chrysin at the doses of 1–20 μM had no cytotoxicity (Figure 1B). Thus, the current experiments employed chrysin in the range of 1–20 μM.

### 2.3. Assays for Nuclear Condensation and DNA Fragmentation

For the measurement of cellular apoptosis were conducted Hoechst 33258 staining and terminal deoxynucleotidyl transferase dUTP nick end labeling (TUNEL). HRMVEC were seeded on a four-well chamber slide and treated with 1–20 μM chrysin in different glucose media. For the Hoechst 33258 staining, cells were fixed with 4% formaldehyde for 15 min. After blocking cells with a 4% FBS for 1 h, HRMVEC were stained with 1 mg/mL of the nuclear dye Hoechst 33258 (Promega Co., Madison, WI, USA) to visualize nuclear condensation and fragmentation under fluorescence microscopy. Images of each slide were taken using an optical microscope system (Axiomager, Zeiss, Oberkochen, Germany).

The DNA fragmentation assay was performed by using a commercial DeadEnd™ Florometric TUNEL kit (Promega Corp.). The cells were fixed with 4% formaldehyde for 25 min at 4 °C and permeabilized with 0.2% Triton X-100. Fluorescein isothiocyanate (FITC)-12-dUTP was added and DNA fragments were detected for 1 h at 37 °C. For the nuclear counterstaining, cells were treated with 4 mg/mL 4′,6-diamidino-2-phenylindole (DAPI, Santa Cruz Biotechnology) in PBS-tween 20 for 10 min. Representative fluorescent mages of each slide were taken and quantified using an optical microscope system (Axiomager). 

### 2.4. Western Blot Analysis 

Western blot analysis was conducted using whole cell lysates prepared from HRMVEC at a density of 3.5 × 10^5^ cells. Mouse eye extracts were also prepared from mice that were supplemented with 10 mg/kg chrysin. Whole cell lysates and mouse eye extracts were prepared in a lysis buffer containing 1 M β-glycerophosphate, 1% β-mercaptoethanol, 0.5 M NaF, 0.1 M Na_3_VO_4_, and protease inhibitor cocktail. Cell lysates and eye tissue extracts containing equal amounts of proteins were electrophoresed on 6%–15% SDS-PAGE and transferred onto a nitrocellulose membrane. Nonspecific binding was blocked with 5% skim milk for 3 h. The membrane was incubated overnight at 4 °C with each primary antibody of target proteins and washed in a Tris buffered saline-Tween 20 (TBS-T) for 10 min. The membrane was then incubated for 1 h with a secondary antibody of goat anti-rabbit IgG, goat anti-mouse IgG, or donkey anti-goat IgG conjugated to HRP. Each target protein level was determined by using immobilon western chemiluminescent HRP substrate (Millipore Corporation, Billerica, MA, USA) and Agfa X-ray film (Agfa-Gevaert, Mortsel, Belgium). Incubation with mouse monoclonal β-actin antibody (Sigma-Aldrich Chemical) was also performed for comparative controls.

### 2.5. In Vivo Animal Experiments

Adult male db/db mice (C57BLKS/+ Lepr^db^ Iar; Jackson Laboratory, Sacramento, CA, USA) and their age-matched non-diabetic db/m littermates (C57BLKS/J; Jackson Laboratory) were used in the current study. Mice were kept on a 12 h light/12 h dark cycle at 23 ± 1 °C with 50% ± 10% relative humidity under specific pathogen-free conditions, fed a standard pellet laboratory chow diet (Cargill Agri Purina, Biopia, Korea) and were supplied by the animal facility of Hallym University. This study employed db/db mice at seven weeks of age because they begin to develop diabetes (hyperglycemia) at the age of 7–8 weeks. The animals were allowed to acclimatize for a week before beginning the feeding experiments. Mice were divided into three subgroups (*n* = 9 for each subgroup). Mice of the first group were non-diabetic db/m control mice, and db/db mice were divided into two subgroups. One group of db/db mice was orally administrated 10 mg/kg chrysin daily for 10 weeks. 

All animal experiments were approved by the Committee on Animal Experimentation of Hallym University and performed in compliance with the University’s Guidelines for the Care and Use of Laboratory Animals (Hallym 2013-125). No mice died and no apparent signs of exhaustion were observed during the experimental period. The 24 h-urine samples were collected during the 10 week-chrysin supplementation, the urine collection was carried out by using metabolic cages. Urinary albumin was measured using Albuwell M ELISA kit (Exocell, Philadephia, PA, USA), according to a manufacturer’s instruction. 

### 2.6. Immunohistochemical Staining

For the immunohistochemical analysis, paraffin-embedded mouse eye tissue sections (5 μm thick) were employed. The sections were placed on glass slides, de-paraffinated and hydrated with xylene and graded alcohol. The sections were pre-incubated in a boiled sodium citrate buffer (10 mM sodium citrate, 0.05% Tween 20, pH 6.0) for antigen retrieval. Specific primary antibody against VEGFR2 or ZO-1 was incubated with the tissue sections overnight. The tissue sections were incubated for 1 h with HRP-conjugated anti-rabbit IgG. For the measurement of VEGFR2 expression, the tissue section was developed with FITC-conjugated anti rabbit IgG. Nuclear staining was performed with 4′,6-diamidino-2-phenylindole (DAPI, Santa Cruz Biotechnology). For the ZO-1 visualization, the sections were visualized with 3,3′-diaminobenzidine (DAB) to produce a brown staining, being counterstained with hematoxylin. The stained tissue sections were examined using an optical Axiomager microscope system (Zeiss, Göttingen, Germany) and five images (400×) were taken for each section. Protein levels of VEGFR2 and ZO-1 were quantified by image analysis program of an optical Axiomager microscope system. 

### 2.7. Retinal Trypsin Digestion Assay

The eyes were removed from mice and fixed in 4% paraformaldehyde solution for 24 h. The eyes were equatorially bisected and the entire retina was removed. The retinas were washed overnight in distilled water and incubated with 3% trypsin in 0.1 M Tris buffer (pH 7.8) for 1 h at 37 °C. Non-vascular tissues were carefully removed and isolated vasculature was air dried on a slide. The retinal vasculature was visualized by staining with H&E and examined using an optical Axiomager microscope system. 

### 2.8. FITC–Dextran Perfused Retinal Flat Mounts

FITC-dextran (20 mg/mL) in phosphate buffered saline was injected into the left ventricle of anesthetized mice. Eyes were enucleated immediately after the perfusion of FITC-dextran and fixed in 4% paraformaldehyde solution for 10 min. Retinas were flat-mounted and examined using an optical Axiomager microscope system. 

### 2.9. Data Analysis

The results are presented as mean ± SEM. Statistical analyses were carried out by using the Statistical Analysis statistical software package version 6.12 (SAS Institute, Cary, NC, USA). Significance was determined by one-way ANOVA, followed by Duncan’s multiple-range test for multiple comparisons. Differences were considered significant at *p* < 0.05. 

## 3. Results

### 3.1. Inhibition of Apoptosis of High Glucose-Induced Retinal Epithelial Cells by Chrysin 

The HRMVEC viability was significantly reduced by exposure of these cells to 33 mM glucose for 48 h, evidenced by the MTT data (Figure 1B). When HRMVEC were treated with 1–20 μM chrysin, the viability was enhanced in a dose-dependent manner. 

To assess endothelial apoptosis under high glucose condition, this study conducted Hoechst 33258 staining and in situ TUNEL assay detecting nuclear condensation and DNA fragmentation, respectively. High glucose promoted nuclear condensation, which was inhibited by treating 1–20 μM chrysin to HRMVEC (Figure 1C). In addition, there was marked DNA fragmentation observed in high glucose-exposed HRMVEC, while green-stained DNA fragments were dose-dependently reduced in ≥1 μM chrysin-treated cells (Figure 1D). These results indicate that submicromolar chrysin alleviated endothelial apoptosis in retina induced by excess glucose.

### 3.2. Inhibitory Effects of Chrysin on Induction of Pro-Angiogenic Proteins 

**Figure 2:**
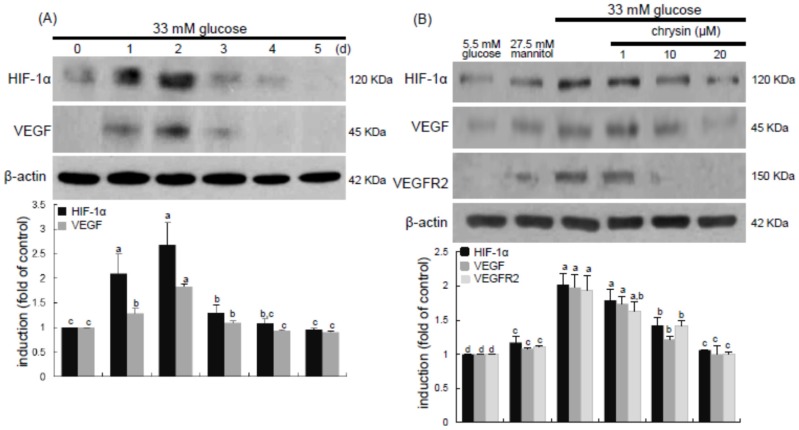
Time course responses of induction of HIF-1α and VEGF by high glucose (**A**) and inhibition of HIF-1α, VEGF, and VEGFR2 by chrysin (**B**). HRMVEC were incubated with 33 mM glucose in the absence and presence of 1–20 μM chrysin up to five days. Cells were also incubated with 5.5 mM glucose and 27.5 mM mannitol as osmotic controls. Cell lysates were subject to Western blot analysis using a primary antibody against HIF-1α, VEGF, or VEGFR2. β-Actin protein was used as an internal control. Bar graphs (mean ± SEM, *n* = 3) in the bottom panels represent densitometric results of upper blot bands. Means not sharing a common letter differ, *p* < 0.05.

Hypoxia has been implicated in the retinal degeneration induced by hyperglycemia, possibly contributing to pathological neovascularization and vascular dystrophies [11,14]. This study examined that chrysin suppressed high glucose-induced angiogenesis through inhibition of hypoxic responses in retina. When HRMVEC were exposed to 33 mM glucose for up to 5 days, the induction of HIF-1α and VEGF proteins was highly enhanced on 2 days post-stimulation with high glucose and, thereafter, vanished (Figure 2A). The enhanced induction of HIF-1α and VEGF was significantly attenuated in cells treated with ≥10 μM chrysin. In addition, the expression of VEGFR2 stimulated by high glucose was dose-dependently diminished by supplementing ≥10 μM chrysin to cells (Figure 2B).

**Figure 3:**
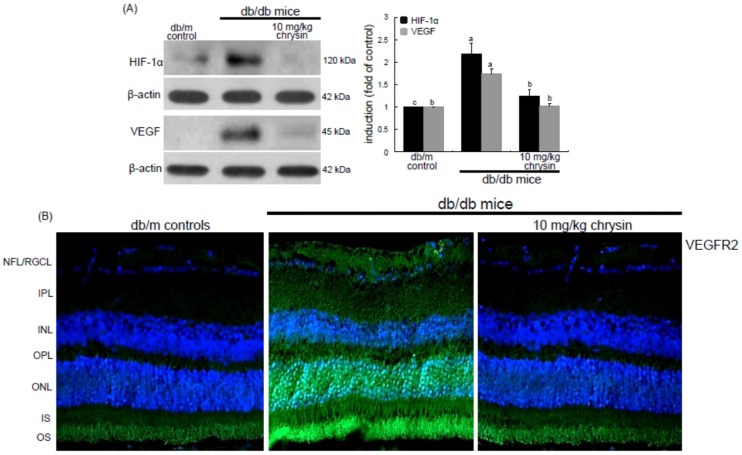
Inhibition of retinal tissue induction of HIF-1α, VEGF and VEGFR2 by chrysin. The db/db mice were orally supplemented with 10 mg/kg chrysin daily for 10 weeks. The db/m mice were introduced as control animals. Mouse retinal tissue extracts were subject to Western blot analysis with a primary antibody against HIF-1α and VEGF (**A**). β-Actin protein was used as an internal control. Bar graphs (mean ± SEM, *n* = 3) in the right panel represent densitometric results of left blot bands. Values not sharing a common letter differ, *p* < 0.05. Histological sections of mouse retina were immunohistochemically stained using a primary antibody of VEGFR2 (**B**). The VEGFR2 was identified as FITC-green staining and the sections were counter-stained with 4′,6-diamidino-2-phenylindole (blue) for the nuclear staining. Magnification: 200×. Retinal layers are labeled as follows: neurofiber layer/ganglion cell layer (NFL/GCL), inner plexiform layer (IPL), inner nuclear layer (INL), outer plexiform layer (OPL), outer nuclear layer (ONL), and photoreceptor inner segment/outer segment (IS/OS).

This study further investigated that retinal angiogenesis was induced in db/db mice, which was encumbered by oral administration of chrysin. The eye tissue levels of HIF-1α and VEGF proteins were elevated in db/db mice (Figure 3A). When db/db mice were orally administrated with 10 mg/kg chrysin for 10 weeks, the tissue levels of these pro-angiogenic proteins decreased. When the FITC-immunohistochemical staining technique for assessing VEGFR2 induction was applied, there was an expected lack of staining in db/m mice (Figure 3B). However, VEGFR2 induction was observed in the overall retinal tissues, with a heavy green staining denoting VEGFR2 in the ganglion cell layer (GCL) and outer nuclear layer (ONL) of db/db mouse retina (Figure 3B). The VEGFR2 was also highly expressed in the photoreceptor outer segment (OS) of diabetic retina. Oral administration of 10 mg/kg chrysin substantially diminished the VEGFR2 staining in the GLC, ONL, and OS (Figure 3B). These observations were consistent with the induction of VEGFR2 in high glucose-stimulated retinal endothelial cells (Figure 2B). Accordingly, chrysin may allay chronic hyperglycemia-induced retinal hypoxia that also causes neovascularization in the retina. 

### 3.3. Elevation of Induction of Endothelial Proteins by Chrysin 

**Figure 4:**
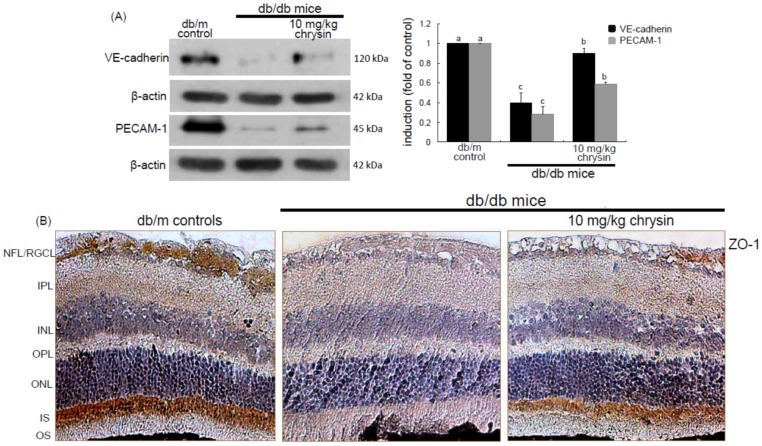
Induction of endothelial junction markers of VE-cadherin, PECAM-1, and ZO-1 by chrysin. The db/db mice were orally supplemented with 10 mg/kg chrysin daily for 10 weeks. The db/m mice were introduced as control animals. Mouse retinal tissue extracts were subject to Western blot analysis with a primary antibody against VE-cadherin and PECAM-1 (**A**). β-Actin protein was used as an internal control. Bar graphs (mean ± SEM, *n* = 3) in the right panel represent densitometric results of left blot bands. Values not sharing a common letter differ, *p* < 0.05. Histological sections of mouse retina were immunohistochemically stained using a primary antibody of ZO-1 (**B**). The ZO-1 expression was identified as 3,3′-diaminobenzidine staining (brown) and the sections were counter-stained with hematoxyline. Magnification: 200×. Retinal layers are labeled as follows: neurofiber layer/ganglion cell layer (NFL/GCL), inner plexiform layer (IPL), inner nuclear layer (INL), outer plexiform layer (OPL), outer nuclear layer (ONL), and photoreceptor inner segment/outer segment (IS/OS).

Hyperglycemia is known to instigate inflammation and oxidative stress leading to vascular dysfunction which increases vascular permeability due to alteration of the blood-retinal barrier (BRB) followed by macular edema and retinal neovascularization [4,32]. The present study determined whether diabetes alters the cellular expression and distribution of the adherens junction protein VE-cadherin, which was inhibited by chrysin. The eye tissue expression of VE-cadherin declined in db/db mice (Figure 4A). In contrast, in 10 mg/kg chrysin-treated db/db mice the VE-cadherin induction was enhanced. In addition, chrysin increased the expression of the endothelial cell-cell adhesion molecule PECAM-1 diminished in diabetic retina (Figure 4A). Furthermore, the ZO-1 localization at inter-endothelial junctions was confirmed by immunohistochemical staining with DAB. The brown staining with DAB disappeared in the GCL and the photoreceptor inner segment (IS) of db/db mouse retina, whereas the immunostaining of ZO-1 was restored in db/db mice treated with 10 mg/kg chrysin (Figure 4B). These results indicate that chrysin may promote the formation of new vessels and prevent the increase in vascular permeability.

### 3.4. Blockade of Retinal Acellular Capillary Formation by Chrysin

**Figure 5:**
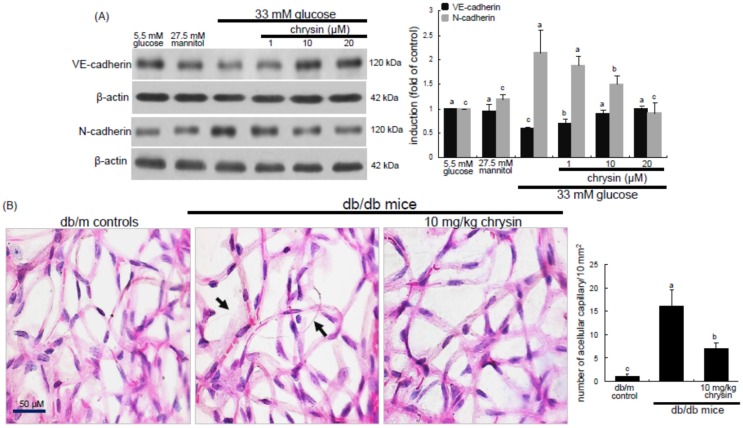
Effect of chrysin on expression of VE-cadherin and *N*-cadherin in high glucose-exposed HRMVEC (**A**) and histopathological changes in trypsin-digested retinal vessels (**B**). HRMVEC were incubated with 33 mM glucose in the absence and presence of 1–20 μM chrysin up to five days. Cells were also incubated with 5.5 mM glucose and 27.5 mM mannitol as osmotic controls. Cell lysates were subject to Western blot analysis using a primary antibody against VE-cadherin or *N*-cadherin (**A**). β-actin protein was used as an internal control. Bar graphs (mean ± SEM, *n* = 3) in the right panels represent densitometric results of left blot bands. The db/db mice were orally treated with 10 mg/kg chrysin for 10 weeks, and db/m mice were introduced as control animals. Retinal vessels were stained with H&E stain (**B**). Acellular capillaries (black arrow) were observed in db/db mice. Magnification: 200×. The number of acellular capillaries was measured to assess the extent of retinopathy. Values in bar graphs (mean ± SEM, *n* = 3) not sharing a common letter differ, *p* < 0.05.

This study examined that chrysin recovered the induction of adherens junction proteins in high glucose-exposed HRMVEC. As expected, the expression of endothelial VE-cadherin was reduced (Figure 5A). However, the treatment of ≥10 μM chrysin enhanced its induction. It has been reported that *N*-cadherin is important for endothelial cell proliferation and motility during angiogenic process [33]. Interestingly, *N*-cadherin was induced in retinal endothelial cells exposed to high glucose (Figure 5A). In chrysin-supplied cells the *N*-cadherin induction decreased, indicating that chrysin attenuated angiogenesis promoted by high glucose.

Acellular capillaries are a pathologic result typical of the loss of both pericytes and endothelial cells in diabetic retina, and their quantification is a useful way to assess the extent of retinopathy [34]. This study further determined whether chrysin blocked the formation of acellular capillaries in trypsin digested retina of db/db mice. H and E staining showed that acellular capillaries were observed in diabetic retinas (Figure 5B). When 10 mg/kg chrysin was treated to diabetic mice, acellular capillaries in retinas disappeared. Diabetic mice treated with chrysin showed no change in the number of acellular capillaries compared with non-diabetic mice. This finding indicates that chrysin may block the formation of acellular capillaries through inhibiting loss of pericytes and endothelial cells via the induction of adherens junction proteins in retinal endothelial cells.

### 3.5. Inhibition of Ang/Tie-2 Receptor Induction by Chrysin 

**Figure 6:**
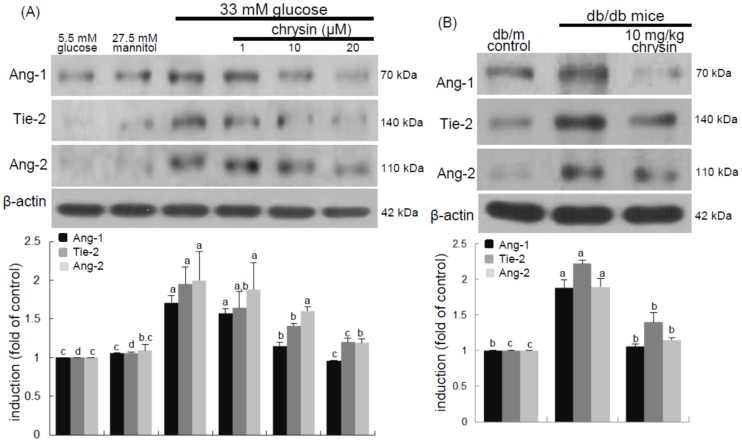
Inhibition of retinal induction of Ang-1, Ang-2, and Tie-2 by chrysin. HRMVEC were cultured in media of 33 mM glucose in the absence and presence of 1–20 μM chrysin for two days (**A**). Cells were also incubated with 5.5 mM glucose and 27.5 mM mannitol as osmotic controls. The db/db mice were orally supplemented with 10 mg/kg chrysin daily for 10 weeks. The db/m mice were introduced as control animals (**B**). HRMVEC lysates and mouse retinal tissue extracts were subject Western blot analysis was conducted for the induction of Ang-1, Ang-2, and Tie-2 using a primary antibody against Ang-1, Ang-2, or Tie-2. β-Actin protein was used as an internal control. Bar graphs in the bottom panel represent densitometric results of upper blot bands. Values (means ± SEM, *n* = 3) not sharing a common letter differ, *p* < 0.05.

Ang-1 activation of different signaling pathways triggered by the Tie-2 tyrosine kinase receptor can promote migration, sprouting, and survival of endothelial cells [21,22]. The current study examined that chrysin blocked high glucose-triggered Ang-Tie-2 signaling in retina. When HRMVEC were exposed to 33 mM glucose for five days, the induction of Ang-1, Ang-2, and Tie-2 proteins was highly enhanced (Figure 6A). The elevated induction of these proteins was significantly attenuated in cells treated with ≥10 μM chrysin. These results showed that chrysin could modulate vessel outgrowth and sprouting leading to the promotion of angiogenesis and vasculogenesis.

This study attempted to confirm that chrysin reduced retinal tissue levels of Ang-1, Ang-2, and Tie-2 proteins elevated in db/db mice. The tissue levels of these proteins were elevated in db/db mouse retina, while oral treatment of db/db mice with 10 mg/kg chrysin for 10 weeks diminished the tissue levels of these proteins (Figure 6B).

### 3.6. Suppressive Effect of Chrysin on New Vessel Formation and Retinal Vascular Leakage

**Figure 7:**
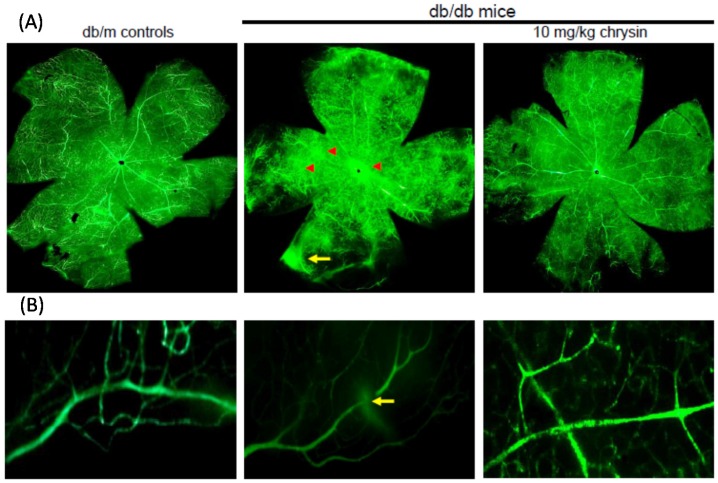
Blockade of new capillary bed formation (**A**) and vascular leakage (**B**) by chrysin. The db/db mice were orally treated with 10 mg/kg chrysin for 10 weeks and db/m mice were introduced as control animals. Typical appearance of ischemic retinopathy was observed by fluorescein-dextran perfused retinal flat-mounts. Retinas were dissected, flat mounted and observed by confocal microscopy. Magnification: 400×.

Hyperglycemia induces retinal neovascularization forming abnormal new blood vessels leading to increased retinal vessel leakage and dystrophies [11,13,14]. To determine the inhibitory effects of chrysin on retinal neovascularization and vascular leakage, FITC-dextran perfusion was performed in a flat mounted mouse retina. In the retinas of db/m mice no vascular abnormalities were observed (Figure 7). In contrast, a significant neovascularization was developed in diabetic retinas and multiple interspersed regions of leakage of the FITC-conjugated dextran appeared in diabetic retinal vessels (yellow arrows). Compared with the db/m controls, there was an increase of neovascular tufts in the diabetic retinas (red arrowheads). Diffuse staining was also observed in retinas of db/db mice. However, fewer neovascular tufts and no diffuse staining were detected in the retinae from db/db mice orally treated with chrysin (Figure 7).

## 4. Discussion

Eight major findings were extracted from this study: (1) hyperglycemia induced a retinal endothelial apoptosis, which was attenuated by chrysin; (2) submicromolar chrysin reduced the induction of HIF-1α, VEGF, and VEGFR2 enhanced in exposed retinal endothelial cells cultured in 33 mM glucose media; (3) The elevated retinal protein levels of HIF-1α, VEGF and VEGFR2 in db/db mice was diminished by oral administration of 10 mg/kg chrysin for 10 weeks; (4) the retinal expression of VE-cadherin, PECAM-1 and ZO-1 declined in db/db mice, whereas there was a substantial increase in the induction of these proteins in chrysin-treated db/db mice; (5) the treatment with ≥10 μM chrysin restored the induction of VE-cadherin and *N*-cadherin diminished in high glucose-exposed retinal endothelial cells; (6) oral administration of chrysin inhibited the formation of acellular capillaries detected in diabetic retinas; (7) treating chrysin attenuated the induction of Ang-1, Ang-2, and Tie-2 proteins in high glucose-exposed retinal endothelial cells and in diabetic retina; and (8) in the FITC-dextran perfused diabetic retina a significant neovascularization and multiple interspersed regions of leakage were observed, which was allayed by orally treating chrysin. These results indicate that chrysin may encumber retinal neovascularization with vascular permeability and BRB breakdown detected in diabetic retina. 

Diabetic ocular manifestations include retinal ischemia, retinal and vitreous morphological changes, and macular edema leading to visual impairment or blindness [1,2,6,8]. Retinal ischemia and hypoxia have been implicated in the retinal degeneration and pathological neovascularization as causative factors occurring due to narrowing or blocked retinal vessels [10,11,13]. In this study, high glucose stimulated the induction of retinal endothelial HIF-1α and the hypoxia-induced factor of VEGF. Consistently, retinal tissue levels of HIF-1α, VEGF, and VEGFR2 were elevated in diabetic eyes. These findings revealed that hyperglycemia appeared to damage retinal vessels, which might result in ischemic retinopathies contributing to retinal neovascularization. The FITC-conjugated dextran perfusion showed that many neovascular tufts were developed in the retinas of diabetic mice. In the current study the pro-angiogenic proteins of Ang-1, Ang-2, and Tie-2 were highly observed in retinal vasculature. One can assume that the overexpression of these Ang proteins may entail vascular dystrophies in diabetic retinas. Chronic hyperglycemia induces retinal cell damage and evokes Ang-2 up-regulation that blocks Tie-2 phosphorylation in retinal endothelial cells, leading to retinal pericyte loss and progressive vasoregression [35]. This study showed the high glucose induced retinal endothelial apoptosis with increased Ang-2 expression.

Many anti-angiogenic drugs have been suggested as potential therapeutic agents in ocular diseases, such as retinopathy of prematurity in children, proliferative diabetic retinopathy in adults, and age-related macular degeneration in the elderly [36,37]. Biologics targeting against HIF/VEGF pathway causing the overexpression of angiogenic genes are the major approach for treatment. Numerous anti-VEGF agents (ranibizumab/lucentis) and HIF inhibitors may be used to achieve the treatment of ocular neovascular diseases [37,38]. However, these agents may cause ocular or systemic side effects after intraocular administration. On the other hand, new drugs at other molecular targets, particularly Ang-2 down-regulation and Tie-2 stabilization, are possible for the treatments of retinal and choroidal vascular diseases [12,21,22]. Potentially detrimental consequences by long-term administration of Ang-1 result in unwanted angiogenesis [21]. In addition, significant efforts in understanding the biological actions and clinical studies involving the applications of these drugs in treating diabetes-associated ocular diseases are limited. Currently, there is growing evidence that inflammatory processes have a substantial role in the pathogenesis of DR. Thus, the use of pharmacological agents with anti-inflammatory activities could be an appealing treatment option for DR. However, there is a need to develop alternative therapies which could halt the progression of DR. 

Although numerous synthetic agents, such as anti-VEGF drugs have been developed as anti-angiogenic agents antagonizing abnormal neovascularization, but their utility has been very limited due to their side effects and poor efficacy. In this scenario, research on natural products with safety profiles in toxicity has been salient. Given the importance of neovascularization or angiogenesis to diverse pathologies, numerous natural compounds have been explored as potential anti-angiogenic drugs [25]. Hence, special attention is imperative to explore the therapeutic potential of these compounds. Natural products may serve as nutraceutical therapeutic interventions and as lead compounds for the specific, efficacious, and affordable drugs with minimal side effects in the eye and its related disorders [25]. Evidence suggests that curcumin and resveratrol may have potential in the treatment of several ocular diseases [39,40]. Scutellarin inhibits retinal angiogenesis through disturbing ROS/HIF-1α/VEGF pathway [27]. In addition, EGCG alleys ocular neovascularization and vascular permeability, congruently accompanying MMP-9 suppression and VEGF activation [26]. This study showed that anti-apoptotic chrysin may antagonize hyperglycemia-induced retinal angiogenesis and neovascularization through suppressing the up-regulation of HIF-1α, VEGF, and VEGFR2 in the retinal vasculature. This study did not investigate the signaling pathways involved in chrysin-related VEGF/VEGFR2-mediated anti-angiogenesis. One investigation shows that chrysin suppresses interleukin-6-induced angiogenesis through down-regulation of JAK1/STAT3/VEGF signaling [41].

Retinal neovascularization accompanies aberrant angiogenesis of retinal vessels, ultimately making these vessels leaky [6,8]. Retinal endothelial cells express Ang-2 as a dominant negative ligand blocking Tie-2 phosphorylation, which induces retinal capillary occlusion and modulates the function of BRB [18,35]. In addition to stimulation of VEGF signaling, the suppression of Tie-2 by Ang-2 is crucial for developing retinal neovascularization and vascular leakage [35]. Thus, the interaction of Ang proteins and Tie-2 may be a potential target for therapies in retinal vascular complications [21]. Modulation of VEGF signaling and Ang-2 expression has been proposed as a tool to correct the cellular mechanisms involved in the pathophysiology of diabetic retinal and choroidal vascular diseases [36]. This study revealed that the up-regulation of both Ang-2 and Tie-2 was inhibited in high glucose-exposed endothelial cells treated with chrysin and retinas of diabetic mice orally administrated chrysin. Accordingly, chrysin may be a therapeutic agent deterring retinal capillary occlusion and BRB breakdown in diabetic retinal and choroidal vascular diseases. In normal retinal capillaries, proper pericyte coverage ensures endothelial cell survival and BRB integrity [35]. Hyperglycemia causes intramural pericyte apoptosis and basement membrane thickening, leading to BRB breakdown and enhanced permeability [3,4,5]. Unfortunately, the current study did not examine the pericyte degeneration in diabetic retina. However, submicromolar chrysin abolished high glucose-induced retinal endothelial apoptosis, which may result in an endothelial cell-induced survival advantage to the pericytes of the vessels. It has been reported that the pericyte-endothelial interaction entices the pericytes toward the endothelial cells [42]. 

Diabetes-associated injury of retinal vessels may leak blood, fluids and exudates into the macula and retina due to BRB breakdown [4]. As stated above, the Ang-2 can influence VEGF, which reduces vascular permeability in diabetic macular edema [18]. This study found that multiple interspersed regions of leakage appeared in diabetic retinal vessels. The Ang-2-VEGF down-regulation appeared to be responsible for the leakage inhibition in diabetic retinal vessels by chrysin. In addition, it was shown that acellular capillaries were formed in diabetic retinas, which was inhibited by oral administration of chrysin. The acellular capillary formation accompanies vascular pericyte depletion and progressive capillary occlusion leading to combined ischemia/hypoxia-induced angiogenesis. Since chrysin inhibited the acellular capillary formation, this could alleviate hypoxia driving the induction of HIF and VEGF. On the other hand, chrysin restored the expression of retinal expression of junction proteins and PECAM-1 abolished by diabetic insults. The endothelial cell-endothelial cell and pericyte-endothelial cell interactions promoted by chrysin may abate retinal vascular permeability and maintain the integrity of the endothelium and optimal BRB function. There is a crosstalk between inflammation and angiogenesis, which contributes to vasopermeability and/or pathological angiogenesis in the DR process [13]. This study did not examine the inhibitory effects of chysin on retinal inflammation. Nevertheless, the anti-inflammatory and antioxidant effects of chrysin would be of potential benefits in pathologies of retinal vascular activation of DR [29,43]. The presence of advanced glycation end products (AGE) in the retinal blood vessel walls contributes towards vascular occlusion and increased permeability of retinal endothelial cells causing vascular leakage [4,44]. Accordingly, chrysin may attenuate VEGF increment and consequent capillary leakage through blunting the accumulation of AGE [45]. 

## 5. Conclusions

This study investigated the capability of chrysin in combating diabetes-mediated retinal neovascularization and microvascular abnormalities in high glucose-exposed retinal endothelial cells and diabetic mice. Chrysin deterred hyperglycemia-elicited induction of the pro-angiogenic proteins of HIF-1α and VEGF involved in retinal neovascularization. Chrysin administration substantially increased the retinal levels of the junction and adhesion proteins of BRB, VE-cadherin, PECAM-1, and ZO-1. In addition, this compound encumbered the formation of acellular capillaries in diabetic retinas possibly due to loss of endothelial adherens junction proteins. Furthermore, chrysin blocked multiple interspersed leakages apparent in retinal vascular beds of diabetic mice. Therefore, chrysin was a therapeutic drug antagonizing retinal injury leading to the retinal neovascularization with increased microvascular abnormalities and BRB permeability in cellular or animal models of diabetic complications in eyes. 

## Abbreviations

AGE	advanced glycation end products
Ang	angiopoietin
BRB	blood retinal barrier
DR	diabetic retinopathy
HIF-1	hypoxia inducible factor-1
*N*-cadherin	neural cadherin
PECAM-1	platelet endothelial cell adhesion molecule-1
ROS	reactive oxygen species
VE-cadherin	vascular endothelial cadherin
VEGF	vascular endothelial growth factor
VEGFR2	VEGF receptor 2
ZO-1	zona occulden-1
